# Topical estrogen application to wounds promotes delayed cutaneous wound healing in 80-week-old female mice

**DOI:** 10.1371/journal.pone.0225880

**Published:** 2019-11-27

**Authors:** Kanae Mukai, Yukari Nakajima, Kimi Asano, Toshio Nakatani

**Affiliations:** 1 Faculty of Health Sciences, Institute of Medical, Pharmaceutical and Health Sciences, Kanazawa University, Kanazawa, Ishikawa, Japan; 2 Department of Clinical Nursing, Graduate Course of Nursing Science, Division of Health Sciences, Graduate School of Medical Sciences, Kanazawa University, Kanazawa, Ishikawa, Japan; University of Pisa, ITALY

## Abstract

Topical estrogen application to wounds is effective in promoting cutaneous wound healing. However, whether it promotes cutaneous wound healing in delayed cutaneous wound healing associated with advanced age remains to be elucidated. This study aimed to evaluate the effect of topical estrogen application to wounds in cutaneous wound healing in 80-week-old female mice. C57BL/6J female mice aged 82–85 and 12 weeks old were submitted to two full-thickness wounds. Mice were divided into four groups: aged group, topical estrogen wound treatment aged group (aged-E), vehicle wound treatment aged group (aged-vehicle), and young group. Wound healing was observed until day 14. In the aged group, wound area ratio (wound area / initial wound area) was significantly higher on days 3–14, ratio of re-epithelialization was significantly lower on day 3 and tended to be lower on day 14, and neutrophil number was significantly higher on day 7 compared with the young group. In contrast, in the aged-E group, wound area ratio was significantly smaller on days 1–14, re-epithelialization ratio was significantly higher on days 3–14, and neutrophil and macrophage number was significantly lower on days 3 and 7 compared with the aged group. These results demonstrate that topical estrogen application to wounds in 80-week-old female mice promoted cutaneous wound healing by reducing wound area and inflammatory response and promoting re-epithelialization.

## Introduction

Due to a complex interaction of clinical and epidemiological factors, the elderly population has rapidly expanded. Between 2015 and 2050, the proportion of individuals aged ≥65 years is estimated to increase from 8.5% to 16.7% of the world’s population [1]. However, increased longevity carries several age-associated physiological changes. Among these changes, functional decline of the skin − one of the largest organs in the body − is pronounced. Skin morphology changes with age, with a decline in dermal thickness, a flattening of the dermo–epidermal junction, and disorganized microcirculation [2–5]. Owing to these morphological and structural changes, skin’s physiological function deteriorates, exhibiting increased dryness and roughness, increased susceptibility to infection, and impaired cutaneous wound healing [6–9].

Cutaneous wound healing is a complex response to injury and involves three major phases: inflammation, proliferation, and remodeling [10]. Additionally, various factors, such as aging, malnutrition, and diseases, are involved in cutaneous wound healing [11]. Since the 1990s, it became clear that cutaneous wound healing is affected by female sex hormones, especially estrogen. Previous studies have reported that postmenopausal women with systemically reduced estrogens show delayed healing, whereas hormone replacement therapy can reverse this delay [12], and that topical estrogen replacement in healthy aged individuals reverses age-associated delayed cutaneous wound healing [13]. Genetically, it has been reported that estrogenic sex hormones play a more important role in human age-associated delayed cutaneous wound healing than intrinsic cellular aging [14]. These studies have attracted attention to estrogens as a potential therapeutic target for promoting cutaneous wound healing. Since then, several animal studies have been performed to clarify estrogen’s effect on cutaneous wound healing. Estrogen administration has been shown to accelerate cutaneous wound healing in 8–12-week-old female mice through suppression of excessive inflammatory cells as neutrophils and macrophages and expression of tumor necrosis factor (TNF)-α [15–21].

Recently, our research group has focused on estrogen administration routes [21]. Slow-release 17β-estradiol (E2) pellet (Innovative Research of America, Sarasota, FL) has been used for subcutaneous administration in several previous studies evaluating the effect of estrogen on cutaneous wound healing [15,16,18,22–24]. In our previous study, E2 gel (L’estrogel 0.06%; Bayer Yakuhin, Osaka, Japan) was applied to the skin [25]. On the other hand, numerous external agents such as honey have been directly applied to wounds for evaluating their effect on cutaneous wound healing [26–29], direct application of estrogen to wounds may also be effective. Our previous study evaluated the effect of topical estrogen application to wounds and compared it with previous treatment methods such as a slow-release E2 pellet and E2 application to the skin. Results suggested that topical estrogen application reduced inflammatory response and promoted angiogenesis and wound contraction to a higher extent than other treatment methods [21]. From this study, it became apparent that topical estrogen application to wounds was more effective in promoting cutaneous wound healing than other methods such as a slow-release E2 pellet and E2 application to the skin.

Our research group has also been interested in the effect of estrogen on cutaneous wound healing upon delayed cutaneous wound healing associated with aging. Our previous studies showed that E2 gel application to the skin promoted cutaneous wound healing in 24- (young) and 40-week-old (mature) female mice by reducing wound area and inflammatory response, and promoting re-epithelialization and wound contraction [30][31]. These studies indicated that E2 gel application to the skin is effective in promoting cutaneous wound healing associated with advanced age. Although our recent study revealed that topical estrogen application to wounds is more effective than E2 gel application to the skin, this study only assessed 12-week-old female mice. Additionally, in the previous study, topical estrogen replacement was applied only before the wounds were made [13]. So, whether topical estrogen application to wounds promotes cutaneous wound healing in cases of delayed cutaneous wound healing associated with advanced age remains to be elucidated. Previous studies have used female mice aged 22–24 months as an aged model [32,33]. On the other hands, tumor incidence tends to rapidly increase after the age of 15 months (incidence rates in various age-groups were: 14–15 months, 6.2%; 20–24 months, 41.3%; and 27–30 months, 61.1%) [34]. So, we chose 80-week-old female mice from the perspective of balancing between life span and tumor incidence rate. Therefore, the present study aimed to assess the effect of topical estrogen application to wounds on cutaneous wound healing in 80-week-old female mice.

## Materials and methods

### Animals

A total of 85 C57BL/6 female mice (Sankyo Lab Service Co., Tokyo, Japan) were used in the experiments. Animals were individually caged in an air-conditioned room at 25.0 ± 2.0°C, with lights on between 08:45 and 20:45 and free access to water and chow. All animal experiments conducted in this study were reviewed and approved by the Animal Experiment and Use Committee of Kanazawa University and performed in accordance with the Guidelines for the Care and Use of Laboratory Animals of Kanazawa University, Japan (AP-153537).

### Wounding

Mice were fed until the age of 82–85 or 12 weeks. Before reaching the age of 82–85 weeks, thirty-three mice died or suffered from disease. At this time, they were anesthetized by inhalational anesthesia using 1.5% isoflurane (Wako, Tokyo, Japan) in 1.5 L O_2_/min through a plastic tube mask. Animals’ dorsum was shaved, and mice were divided into four groups: aged group, topical estrogen wound treatment aged group (aged-E), vehicle wound treatment aged group (aged-vehicle), and young group. One day after shaving, mice dorsum was disinfected with 70% ethanol, then two circular full-thickness skin wounds (4 mm in diameter) including the panniculus carnosus muscle were made on both sides of mice dorsum using a Kai sterile disposable biopsy punch (Kai Industries Co. Ltd., Gifu, Japan) under inhalational anesthesia. Wounds were covered with a hydrocolloid dressing (Tegaderm; 3M Health Care, Tokyo, Japan) to maintain a moist environment, and mice were wrapped with sticky bandages (Skinergate^™^; Nichiban, Tokyo, Japan), which were replaced every day in according to our previous studies [21,25,30,31].

### Exogenous estrogen administration

Estradiol benzoate (OVAHORMON^®^INJECTION; ASKA Pharmaceutical Co. Ltd., Tokyo, Japan) was applied to wounds at 0.75 μg/g/day every day following wounding procedures under inhalational anesthesia [21]. In brief, estradiol benzoate was applied to cover the wound surface after cleaning the wounds with normal saline. Estradiol benzoate was diluted at 0.75 μg/g in sesame oil (Wako Pure Chemical Industries Ltd., Tokyo, Japan). The sesame oil vehicle was applied at the same dose as estradiol benzoate to wounds every day following wounding.

### Macroscopic observations

The day on which animals were wounded was designated as day 0. The process of wound healing was observed until day 14 under inhalational anesthesia. Wound edges were traced on polypropylene sheets, and images were captured every day. Traces on the sheets were captured with a scanner onto a personal computer using Adobe Photoshop Elements (APE) 11.0 (Adobe System Inc., Tokyo, Japan), and wound areas were calculated using ImageJ image analysis software (National Institutes of Health, Bethesda, Maryland, USA) according to our previous studies [21,25,30,31]. Wound area was defined as the wound area ratio each day since the initial wound area on day 0, when wound was made [21,25,30,31]. The term “rapidly” was defined as decrease in the wound area ratio until it became flat and the term “gradually” was defined as decrease in the wound area ratio after it became flat.

### Tissue collection

Mice were euthanized through a pentobarbital sodium overdose intraperitoneally administered on days 3, 7, and 14 post-wounding (day 3: 3 mice per groups; days 7 and 14: 5 mice per groups). Wound and surrounding intact skin (epidermis, dermis and subcutaneous tissue) which area was about 10% of the wound were harvested (day 3: 6 wounds per group; days 7 and 14: 10 wounds per groups), and each wound sample were bisected at the wound center. Half of each wound sample was embedded in tissue-Tek OCT (Sakura Finetek, Japan) and frozen in liquid nitrogen then stored at -20°C prior to fixing for histology, and the remaining half was snap-frozen in liquid nitrogen and stored at −80°C prior to RNA isolation.

### Immunohistological staining

Five-μm-thick ice sections were stained with hematoxylin and eosin staining or subjected to immunohistology with an anti-α smooth muscle actin (α-SMA) antibody (ab5694, Abcam Japan, Tokyo, Japan), an anti-neutrophil antibody (ab2557, Abcam Japan, Tokyo, Japan), and an anti-Mac-3 antibody (550292, BD Pharmingen, Tokyo, Japan). To detect primary antibodies, sections for the anti-α-SMA antibody were incubated with the Dako Envision+ system HRP-labeled polymer anti-rabbit (ready to use) (Dako North America, California, USA) and sections for the anti-neutrophil and anti-Mac-3 antibodies were incubated with polyclonal rabbit anti-rat immunoglobulins/HRP (Dako North America, California, USA). These immunohistological staining was performed in accordance with our previous studies [21,25,30,31]. Negative control slides were obtained by omitting each primary antibody.

### Microscopic observations

Images were imported onto a computer using a digital microscopic camera (DP2-BSW Olympus, Japan). Measurements of re-epithelialization proportions (re-epithelialization length/wound length) on days 3, 7 and 14 were performed using DP2-BSW software. Each positively stained cell (neutrophil and macrophage) on days 3 and 7 was counted at five wound sites using ImageJ image analysis software with a 40× objective, according to our previous studies [21,25]. The five sites’ total area was calculated on DP2-BSW monitor, and total neutrophil and macrophage numbers in the five wound sites were divided by the whole five sites’ total area. Measurements of the ratio of myofibroblasts (myofibroblast pixels/total wound pixels) on days 7 and 14 were performed using APE software, according to our previous studies [21,25,30,31].

### RNA isolation and real-time polymerase chain reaction (PCR)

RNA was isolated from the whole-wound homogenate using PureLink RNA Mini Kit (ThermoFisher Scientific K.K., MA, USA). cDNA was transcribed from 1μg of RNA using the PrimeScript^™^ RT reagent Kit with gDNA Eraser (TAKARA Bio Inc., Shiga, Japan). Quantitative real-time PCR was performed using TB Green^™^ Premix Ex Taq^™^ Ⅱ (TAKARA Bio Inc., Shiga, Japan) and an AriaMx Real-Time PCR system (Agilent Technologies Inc., CA, USA). Each sample was serially diluted over three orders of magnitude, and the expression levels normalized to those of *Gapdh* housekeeping gene. Relative expression ratios were determined in relation to a control sample (young group). Primer sequences were as follows. *Tnf-a*: F, 5′-ACGTCGTAGCAAACCACCAA-3′; R, 5′-AAGGTACAACCCATCGGCTG-3′, *Il-6*: F, 5′-CCGGAGAGGAGACTTCACAG-3′; R, 5′-TCCACGATTTCCCAGAGAAC-3′, and *Gapdh*: F, 5′-TGATGGGTGTGAACCACGAG-3′; R, 5′-GGCATGGACTGTGGTCATGA-3′.

### Statistical analysis

Data was expressed as mean ± standard error of the mean (SEM) and analyzed using JMP^®^ 12.1.0 (SAS Institute Inc., Cary, NC, USA). Mean comparisons among multiple groups were performed using one-way ANOVA followed by post hoc pairwise comparisons using the Tukey–Kramer multiple comparison test. P *<*0.05 was considered significant.

## Results

### Wound area

In the young group, wound areas increased for 3 days (wound area to initial wound area ratio on day 3: 1.18 ± 0.04) and then rapidly decreased until day 9 (0.26 ± 0.05), after which gradually decreased until day 14 (0.06 ± 0.02). In the aged group, wound areas increased for 3 days (1.57 ± 0.11) and then decreased until day 14 (0.35 ± 0.09). In contrast, in the aged-E group wound areas did not increase and instead rapidly decreased until day 5 (0.34 ± 0.03), after which they gradually decreased until day 14 (0.06 ± 0.01). Additionally, in the aged-vehicle group, wound areas increased for 2 days (on day 2: 0.95 ± 0.17) and then rapidly decreased until day 5 (0.30 ± 0.04), after which they gradually decreased until day 14 (0.10 ± 0.02).

**Fig 1:**
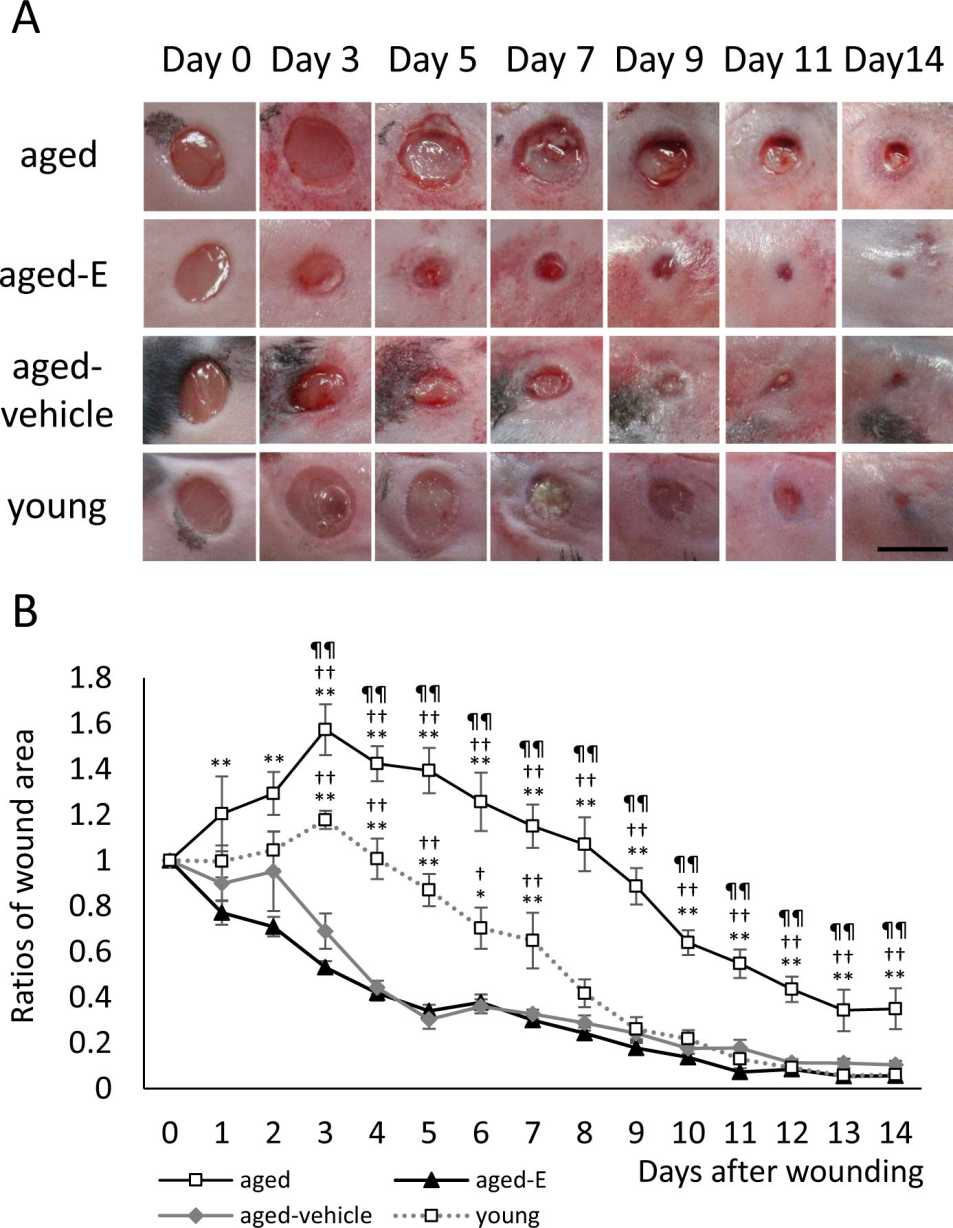
Macroscopic wound healing. (A) Wounds with a 4 mm-diameter were inflicted, and healing was recorded by image capture. Scale bar, 5 mm. (B) Wound area ratios in relation to initial area on day 0 are depicted as line graphs for each day. Values are expressed as mean ± SEM, n = 6–10 wounds per groups, ANOVA, Tukey–Kramer **p<0.01 vs. aged-E group; ^††^p<0.01 vs. aged-vehicle group and ^¶¶^p<0.01 vs. young group.

Wound area ratio was significantly higher in the aged group compared with the young group on days 3–14 (p<0.01). In contrast, wound area ratios were significantly lower in the aged-E and aged-vehicle groups compared with the aged group on days 1–14 (p<0.01) and days 3–14 (p<0.01), respectively. Wound area ratios were also significantly lower in the aged-E and aged-vehicle groups compared with the young group on days 3–7 (p<0.01 and p<0.05, respectively). There were no significant differences between aged-E and aged-vehicle groups (Fig 1A and 1B).

### Re-epithelialization and wound contraction

**Fig 2:**
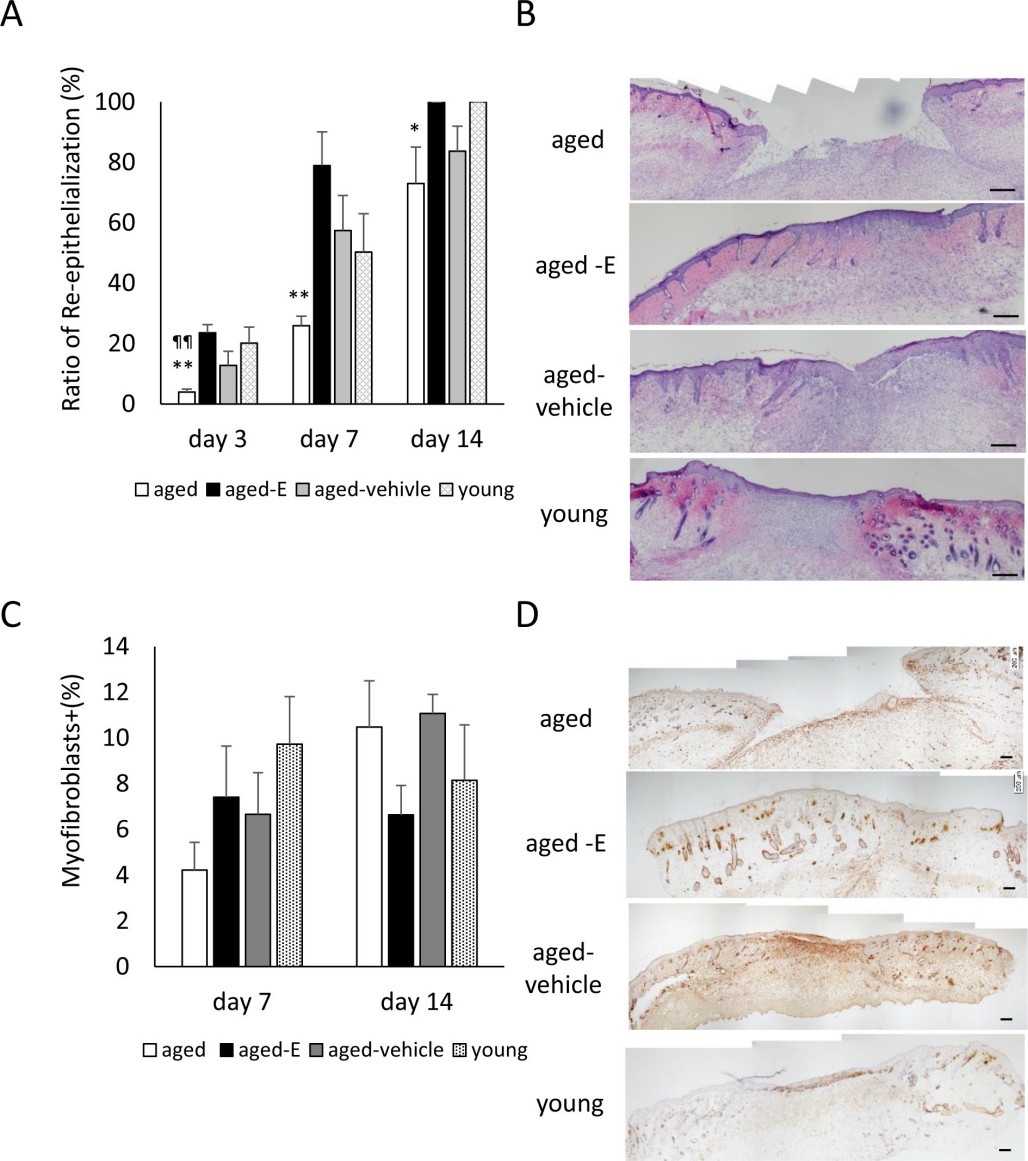
Re-epithelialization and wound contraction. Box graphs show (A) ratio of re-epithelialization (%) and (C) ratio of myofibroblasts (%). (B) HE staining (scale bar = 200 μm) and (D) myofibroblasts stained with anti-α-SMA antibody (scale bar = 200 μm) were observed in granulation tissue on day 14. Values are expressed as mean + SEM, n = 4–8 wounds for each group, ANOVA, Tukey–Kramer *p<0.05 and **p<0.01: vs. aged-E group; ^¶^p<0.05 and ^¶¶^p<0.01 vs. young group.

In the young group, new epithelium gradually extended from the wound edges and completely covered the whole wound by day 14. In contrast, in the aged group, although new epithelium gradually extended from wound edges, it did not completely cover the whole wound on day 14. Following topical estrogen wound treatment, new epithelium rapidly developed from wound edges and completely covered the whole wound by day 14. In the aged-vehicle group, new epithelium developed from wound edges, but it did not completely cover the whole wound until day 14. The ratio of re-epithelialization was significantly lower in the aged compared with the young group on day 3 (p <0.01) and tended to be lower in the aged compared with the young group on day 14 (p = 0.0574). Re-epithelialization ratio was significantly higher in the aged-E than in the aged group on days 3–14 (p <0.05) (Fig 2A and 2B).

In the young group, anti-α SMA antibody-positive myofibroblasts (differentiated fibroblasts with strong contractility) were observed with bridge-like structure which was the appearance of myofibroblasts connecting the wound edges across the wound on day 7, and subsequently decreased in number until day 14. In contrast, in the aged group, anti-α SMA antibody-positive myofibroblasts appeared at the wound edges and granulation tissue on days 7 and 14. However, they were not connected linearly. Following topical estrogen wound treatment, anti-α SMA antibody-positive myofibroblasts were observed with bridge-like structures across the wound on day 7, and subsequently decreased in number until day 14. In the aged-vehicle group, anti-α SMA antibody-positive myofibroblasts were observed in granulation tissue on day 7, persisting in granulation tissue while maintaining a bridge-like structure on day 14. No significant differences were found among groups (Fig 2C and 2D).

### Neutrophils, macrophages, TNF-α, and IL-6

**Fig 3:**
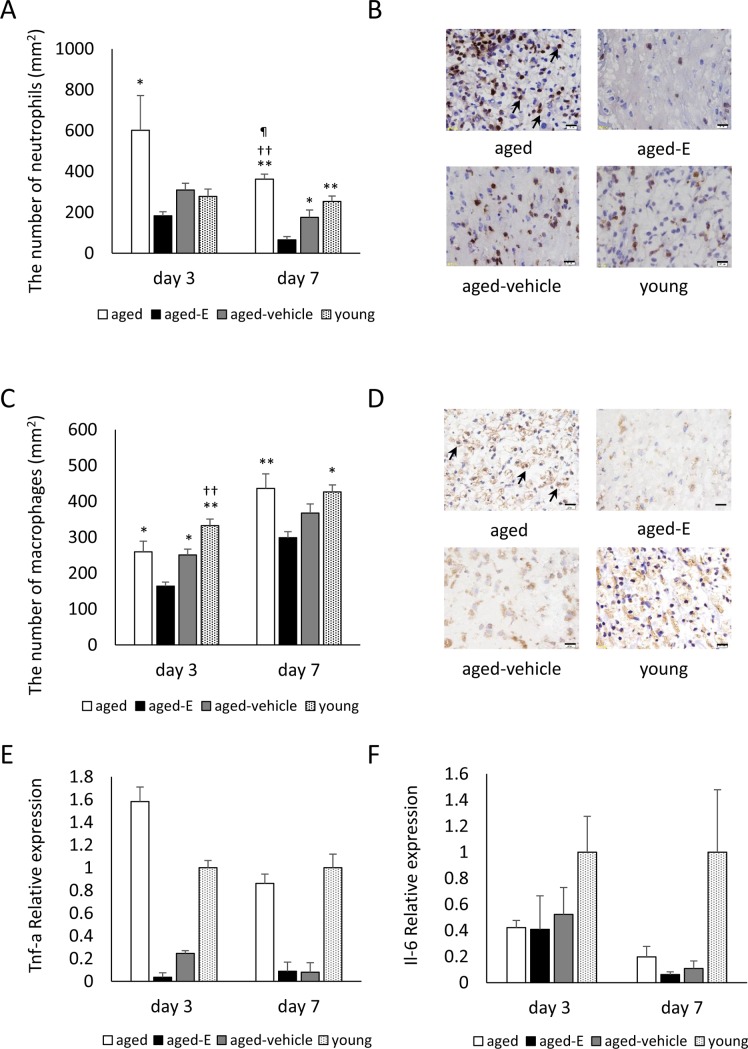
Neutrophils, macrophages, and relative *Tnf-a* and *Il-6* expression. Box graphs show (A) number of neutrophils per mm^2^ and (C) number of macrophages per mm^2^. (B) Neutrophils (arrows) stained with anti-neutrophil antibody (scale bar = 20 μm) and (D) macrophages (arrows) stained with anti-Mac-3 antibody (scale bar = 20 μm) were observed in wound tissue on day 7. Box graphs show (E) relative *Tnf-a* expression and (F) relative *Il-6* expression. Values are expressed as mean + SEM, n = 5–6 wounds for each group (A−D) and n = 3 wounds for each group (E and F), ANOVA, Tukey–Kramer *p<0.05 and **p<0.01 vs. aged-E group; ^††^p<0.01 vs. aged-vehicle group; ^¶^p<0.05 vs. young group.

Large neutrophil numbers were observed in wounds of all groups on day 3, particularly in the aged group. Numbers then decreased until day 7, particularly in the aged-E group. Neutrophil number was significantly higher in the aged compared with the aged-E group on days 3 and 7 (p<0.05) and with the young and aged-vehicle groups on day 7 (p<0.05 and p<0.01, respectively). In addition, neutrophil number was significantly lower in the aged-E compared with the young and aged-vehicle groups on day 7 (p<0.05 and p<0.01, respectively) (Fig 3A and 3B).

Macrophages were observed in wounds of all groups on day 3 and increased until day 7. Macrophage numbers were significantly lower in the aged-E compared with the other three groups on day 3 (p<0.05) and with the aged and young groups on day 7 (p<0.01 and p<005, respectively). In addition, macrophage number was significantly lower in the aged-vehicle than in the young group on day 3 (p<0.05) (Fig 3C and 3D).

In the aged group, relative *Tnf-a* expression was high compared with the young group on day 3, although not significantly different. Relative *Tnf-a* expression in the aged-E and aged-vehicle groups was low compared with the young group on days 3 and 7, but not significantly different (Fig 3E).

In the aged, aged-E, and aged-vehicle groups, relative *Il-6* expression was low compared with the young group on days 3 and 7, although not significantly different (Fig 3F).

## Discussion

Since the 1990s, estrogen has attracted increasing attention as a therapeutic target for cutaneous wound healing [12,15,18,20,23,35]. In our previous study, we revealed that topical estrogen application to wounds was more effective in promoting cutaneous wound healing compared with other standard methods [21]. However, in the previous study, topical estrogen replacement therapy was only applied before wounds were made [13]; therefore, whether topical estrogen application to wounds promotes cutaneous wound healing upon delayed cutaneous wound healing due to advanced age remains unclear. Therefore, the present study aimed to examine the effect of topical estrogen application to wounds on cutaneous wound healing in 80-week-old female mice. Results revealed that topical estrogen application to wounds promotes cutaneous wound healing in 80-week-old female mice showing delayed cutaneous wound healing; however the between-group differences with respect to cutaneous wound healing parameters were not statistically significant between application of topical estrogen and sesame oil as vehicle.

In the aged group, wound area expanded for 3 days in the inflammatory phase and remained large throughout the whole period compared with the young group. In contrast, expanded wound area in the aged group was suppressed following topical estrogen application to wounds. Topical estrogen application to wounds reduced their area compared with that of the young group on days 3–7 post-wounding in the inflammatory phase. Our previous findings demonstrated that wound area expanded in the inflammatory phase and gradually decreased afterwards [21,25,30,31]. So results hence seemed to show that topical estrogen application to wounds is effective in reducing the inflammatory response in delayed cutaneous wound healing associated with advanced age. In support of these results, the present study compared neutrophil and macrophage numbers in wounds. In the inflammatory phase, bacteria are removed and a suitable environment is created for eliciting tissue repair and regeneration [36–38]. Neutrophils and macrophages are believed to play a major role in the inflammatory phase. Neutrophils prevent infection through their phagocytic ability as the first line of defense and release pro-inflammatory cytokines [38,39], subsequently, days later, macrophages also exhibit phagocytic ability and release pro-inflammatory cytokines [40,41]. In the present study, a large neutrophil number was observed in wounds on day 3 in the aged group compared with the young group, which persisted on day 7. Previous studies have documented a marked early increase in the neutrophil number in the aged and a less pronounced peak in the wounds of young subjects [42]; in addition, advanced age has been shown to be associated with impaired neutrophil chemotaxis [43–45]. Therefore, it appears that aged mice have impaired inflammatory response with persistence of neutrophils in wounds. In contrast, the increased neutrophil number and survival in the aged group was reduced following topical estrogen application. Several previous studies have reported reduction in the number of neutrophils upon estrogen administration in wounds of young mice (age; 8–12 weeks) [16,17,23] as well as in mice aged 24 weeks and 40 weeks [30,31]. In the present study, the number of macrophages in wounds was also significantly reduced following topical estrogen application compared with that in young and aged groups. In previous studies, estrogen administration was shown to decrease the number of macrophages in wounds in young (age: 8–12 weeks) [16–19,23]. Therefore, these results suggest that the altered inflammatory response with cellularity in aged mice improved with topical estrogen application to wounds. We also evaluated the relative expression of pro-inflammatory cytokines in this study. The crucial role of TNF-α and IL-6 in the pathophysiology of specific inflammatory conditions is well documented [40,41,46]; in addition, delayed cutaneous wounds were shown to exhibit raised local and systemic levels of TNF-α and IL-6 in aged [47,48]. Although no significant difference was observed, the relative *Tnf-a* expression in the aged group was higher than that in the young group, and was suppressed after topical application of estrogen. On the other hand, although no significant difference was observed, the relative *Il-6* expression in aged group was lower than that in the young group, and this expression was also low in the aged-E group compared with the young group. Previous studies reported that elevated *IL-6* is associated with inflammation in aged and senescent cells [49,50]; in addition, *IL-6* expression was reduced in aged keratinocytes following wounding compared with young keratinocytes [32]. These results suggest that the effect of aging on secretion of inflammatory cytokines may vary. Further research is required to confirm this unclear phenomenon.

When the inflammatory response smoothly ends, wound closure involves epithelial stretching and wound contraction [10]. In the present study, new epithelium did not completely cover wound surface until day 14 in the aged group, and anti-α SMA antibody-positive myofibroblasts had not formed a bridge-like structure on day 14. In contrast, following topical estrogen application to wounds, new epithelium had completely covered wound surface by day 14, and anti-α SMA antibody-positive myofibroblasts were observed in the granulation tissue with forming bridge-like structures across the wound on day 7, and subsequently decreased until day 14 (However, there were no significant differences). Previous studies have reported that estrogen promotes re-epithelialization in 8–12-week-old young mice [16,17,23] and in 24- and 40-week-old mice [30,31]. Therefore, our results together with previous findings show that topical estrogen application to wounds effectively promotes re-epithelialization in delayed cutaneous wound healing due to advanced age, although not significantly affecting wound contraction. Keyes et al. recently reported that re-epithelialization in aged skin was impaired through activation of transcription (STAT) 3 signaling [32] and IL-6 treatment which was a ligand of STAT3-signaling improved keratinocyte outgrowth [32]. So, it was thought that re-epithelialization was promoted by estrogen application though IL-6/STAT-3 signaling. However in this study, relative *Il-6* expression was low in the aged-E group and did not reach the same level as observed in young group. Campbell et al. reported that estrogen promotes mouse keratinocyte migration through estrogen receptor (ER)-β [23]. Therefore, we believe that estrogen may promote re-epithelialization through ER-β rather than STAT3-mediated signaling in the setting of delayed cutaneous wound healing associated with advanced age. Further research is required to confirm this hypothesis.

In the present study, sesame oil was used as a treatment vehicle, as it was predicted that cutaneous wound healing in aged and aged-vehicle groups can be similar. However, contrarily to initial expectations, sesame oil promoted cutaneous wound healing to some extent compared with the observed healing in the aged group with reduced wound area and neutrophil number. As no differences were observed in the healing parameters between estrogen and vehicle, the effect of the vehicle reached that of estrogen. Several previous studies have reported the effectiveness of this oil in promoting cutaneous wound healing in young animals [51–54], and the present results suggest that sesame oil as a treatment vehicle somewhat promotes cutaneous wound healing in aged mice. Therefore, further research is required in the near future to clarify the mechanisms underlying cutaneous wound healing promoted by topical sesame oil application to wounds in aged mice.

In conclusion, the present study showed that topical estrogen application to wounds with delayed cutaneous healing due to advanced age reduced wound area and inflammatory response and promoted re-epithelialization. Topical estrogen application to wounds therefore promoted cutaneous wound healing in 80-week-old female mice showing delayed cutaneous wound healing. These results will potentially be beneficial in wound care for elderly women.

## References

[1] He W, Goodkind D, Kowal P. An Aging World: 2015. International Population Reports. U.S. Government Printing Office, Washington DC 2016:1–165.

[2] Montagna W, Carlisle K. Structural changes in ageing skin. Br J Dermatol. 1990;122 Suppl: 61–70. 10.1111/j.1365-2133.1990.tb16127.x 2186788

[3] Fenske NA, Lober CW. Structural and functional changes of normal aging skin. J Am Acad Dermatol. 1986;15:571–585. 10.1016/s0190-9622(86)70208-9 3534008

[4] Kurban RS, Bhawan J. Histologic changes in skin associated with aging. J Dermatol Surg Oncol. 1990;16: 908–914. 10.1111/j.1524-4725.1990.tb01554.x 2229632

[5] Richard S, Querleux B, Bittoun J, Jolivet O, Idy-Peretti I, de Lacharriere O, et al Characterization of the skin in vivo by high resolution magnetic resonance imaging: water behavior and age-related effects. J Invest Dermatol. 1993;100: 705–709. 10.1111/1523-1747.ep12472356 8388010

[6] Ashcroft GS, Mills SJ, Ashworth JJ. Ageing and wound healing. Biogerontology. 2002; 337–345. 10.1023/a:1021399228395 12510172

[7] Kenyon CJ. The genetics of ageing. Nature. 2010;464: 504–512. 10.1038/nature08980 20336132

[8] Velarde MC, Demaria M, Melov S, Campisi J. Pleiotropic age-dependent effects of mitochondrial dysfunction on epidermal stem cells. Proc Natl Acad Sci USA. 2015;112: 10407–10412. 10.1073/pnas.1505675112 26240345 PMC4547253

[9] Ashcroft GS, Horan M a, Ferguson MW. The effects of ageing on cutaneous wound healing in mammals. J Anat. 1995;187 (Pt 1): 1–26. 7591970 PMC1167345

[10] Shaw TJ, Martin P. Wound repair at a glance. J Cell Sci. 2009;122: 3209–3213. 10.1242/jcs.031187 19726630 PMC2736861

[11] Sgonc R, Gruber J. Age-related aspects of cutaneous wound healing: A mini-review. Gerontology. 2013;59: 159–164. 10.1159/000342344 23108154

[12] Ashcroft GS, Dodsworth J, van Boxtel E, Tarnuzzer RW, Horan MA, Schultz GS, Ferguson FM. Estrogen accelerates cutaneous wound healing associated with an increase in TGF-β1 levels. Nat Med. 1997;3: 1209–1215. 9359694 10.1038/nm1197-1209

[13] Ashcroft GS, Greenwell-Wild T, Horan MA, Wahl SM, Ferguson MW. Topical estrogen accelerates cutaneous wound healing in aged humans associated with an altered inflammatory response. Am J Pathol. 1999;155: 1137–1146. 10.1016/S0002-9440(10)65217-0 10514397 PMC1867002

[14] Hardman MJ, Ashcroft GS. Estrogen, not intrinsic aging, is the major regulator of delayed human wound healing in the elderly. Genome Biol. 2008;9 10.1186/gb-2008-9-5-r80 18477406 PMC2441466

[15] Ashcroft GS, Mills SJ, Lei K, Gibbons L, Jeong MJ, Taniguchi M, et al Estrogen modulates cutaneous wound healing by downregulating macrophage migration inhibitory factor. J Clin Invest. 2003;111: 1309–1318. 10.1172/JCI16288 12727922 PMC154440

[16] Emmerson E, Campbell L, Ashcroft GS, Hardman MJ. The phytoestrogen genistein promotes wound healing by multiple independent mechanisms. Mol Cell Endocrinol. 2010;321: 184–193. 10.1016/j.mce.2010.02.026 20193736

[17] Hardman MJ, Emmerson E, Campbell L, Ashcroft GS. Selective estrogen receptor modulators accelerate cutaneous wound healing in ovariectomized female mice. Endocrinology. 2008;149: 551–557. 10.1210/en.2007-1042 17974625

[18] Routley CE, Ashcroft GS. Effect of estrogen and progesterone on macrophage activation during wound healing. Wound Repair Regen. 2009;17: 42–50. 10.1111/j.1524-475X.2008.00440.x 19152650

[19] Brufani M, Ceccacci F, Filocamo L, Garofalo B, Joudioux R, Bella A La, et al Novel locally active estrogens accelerate cutaneous wound healing. A preliminary study. Mol Pharm. 2009;6: 543–556. 10.1021/mp800206b 19718805

[20] Emmerson E, Campbell L, Ashcroft GS, Hardman MJ. Unique and synergistic roles for 17β-estradiol and macrophage migration inhibitory factor during cutaneous wound closure are cell type specific. Endocrinology. 2009;150: 2749–2757. 10.1210/en.2008-1569 19196797

[21] Mukai K, Urai T, Asano K, Nakajima Y, Nakatani T. Evaluation of effects of topical estradiol benzoate application on cutaneous wound healing in ovariectomized female mice. PLoS One. 2016;11:e0163560 10.1371/journal.pone.0163560 27658263 PMC5033238

[22] Gilliver SC, Ruckshanthi JPD, Hardman MJ, Nakayama T, Ashcroft GS. Sex dimorphism in wound healing: The roles of sex steroids and macrophage migration inhibitory factor. Endocrinology. 2008;149: 5747–5757. 10.1210/en.2008-0355 18653719

[23] Campbell L, Emmerson E, Davies F, Gilliver SC, Krust A, Chambon P, et al Estrogen promotes cutaneous wound healing via estrogen receptor β independent of its antiinflammatory activities. J Exp Med. 2010;207: 1825–1833. 10.1084/jem.20100500 20733032 PMC2931162

[24] Gilliver SC, Emmerson E, Chambon P, Hardman MJ, Ashcroft GS. 17B-Estradiol Inhibits Wound Healing in Male Mice Via Estrogen Receptor-A. Am J Pathol. 2010;176: 2707–2721. 10.2353/ajpath.2010.090432 20448060 PMC2877833

[25] Mukai K, Nakatani T, Sugama J, Komatsu E, Nakajima Y, Nasruddin, et al The Effect of 17β-Estradiol on Cutaneous Wound Healing in Protein-Malnourished Ovariectomized Female Mouse Model. PLoS One. 2014;9: e115564 10.1371/journal.pone.0115564 25518000 PMC4269450

[26] Hozzein WN, Badr G, Al Ghamdi AA, Sayed A, Al-Waili NS, Garraud O. Topical application of propolis enhances cutaneous wound healing by promoting TGF-beta/Smad-mediated collagen production in a streptozotocin-induced type I diabetic mouse model. Cell Physiol Biochem. 2015;37: 940–954. 10.1159/000430221 26381245

[27] Naraginti S, Kumari PL, Das RK, Sivakumar A, Patil SH, Andhalkar VV. Amelioration of excision wounds by topical application of green synthesized, formulated silver and gold nanoparticles in albino Wistar rats. Mater Sci Eng C Mater Biol Appl. 2016;62: 293–300. 10.1016/j.msec.2016.01.069 26952426

[28] Ahn J, Kim SG, Kim MK, Kim DW, Lee JH, Seok H, et al Topical delivery of 4-hexylresorcinol promotes wound healing via tumor necrosis factor-α suppression. Burns. 2016;42: 1534–1541. 10.1016/j.burns.2016.04.016 27198070

[29] Farzadinia P, Jofreh N, Khatamsaz S, Movahed A, Akbarzadeh S, Mohammadi M, et al Anti-inflammatory and Wound Healing Activities of Aloe vera, Honey and Milk Ointment on Second-Degree Burns in Rats. Int J Low Extrem Wounds. 2016;15: 241–247. 10.1177/1534734616645031 27217089

[30] Mukai K, Nakajima Y, Urai T, Komatsu E, Takata K, Miyasaka Y, et al The Effect of 17β-Estradiol on Cutaneous Wound Healing in 24-Week Ovariectomized Female Mice. J Horm. 2014; Article ID 234632, 8 pages. 10.1371/journal.pone.0115564 25518000 PMC4269450

[31] Mukai K, Nakajima Y, Urai T, Komatsu E, Nasruddin, Sugama J, et al 17B-Estradiol Administration Promotes Delayed Cutaneous Wound Healing in 40-Week Ovariectomised Female Mice. Int Wound J. 2016;13: 636–644. 10.1111/iwj.12336 25132513 PMC7949953

[32] Keyes BE, Liu S, Asare A, Naik S, Levorse J, Polak L, et al Impaired Epidermal to Dendritic T Cell Signaling Slows Wound Repair in Aged Skin. Cell. 2016;167: 1323–1338.e14. 10.1016/j.cell.2016.10.052 27863246 PMC5364946

[33] Ashcroft GS, Horan MA, Ferguson MWJ. Aging Is Associated with Reduced Deposition of Specific Extracellular Matrix Components, an Upregulation of Angiogenesis, and an Altered Inflammatory Response in a Murine Incisional Wound Healing Model. J Invest Dermatol. 1997;108: 430–437. 10.1111/1523-1747.ep12289705 9077470

[34] Felicio LS, Nelson JF, Finch CE. Spontaneous pituitary tumorigenesis and plasma oestradiol in ageing female C57BL/6J mice. Exp Gerontol. 1980;15: 139–143. 10.1016/0531-5565(80)90085-6 7190097

[35] Campbell L, Emmerson E, Williams H, Saville CR, Krust A, Chambon P et al Estrogen receptor-alpha promotes alternative macrophage activation during cutaneous repair. J Invest Dermatol. 2014;134(9): 2447–2457. 10.1038/jid.2014.175 24769859

[36] Abbott RE, Corral CJ, MacIvor DM, Lin X, Ley TJ, Mustoe TA. Augmented inflammatory responses and altered wound healing in cathepsin G-deficient mice. Arch Surg. 1998;133: 1002–1006. 10.1001/archsurg.133.9.1002 9749856

[37] Hübner G, Brauchle M, Smola H, Madlener M, Fässler R, Werner S. Differential regulation of pro-inflammatory cytokines during wound healing in normal and glucocorticoid-treated mice. Cytokine. 1996;8: 548–556. 10.1006/cyto.1996.0074 8891436

[38] Mercado AM, Quan N, Padgett DA, Sheridan JF, Marucha PT. Restraint stress alters the expression of interleukin-1 and keratinocyte growth factor at the wound site: an in situ hybridization study. J Neuroimmunol. 2002;129(1–2):74–83. 10.1016/s0165-5728(02)00174-1 12161023

[39] Delgado A V, Mcmanus AT, Chambers JP. Production of Tumor Necrosis Factor-alpha, Interleukin 1-beta, Interleukin 2, and Interleukin 6 by rat leukocyte subpopulations after exposure to Substance P. Neuropeptides. 2003;37(6):355–361. 10.1016/j.npep.2003.09.005 14698678

[40] Feldmann M, Williams RO, Paleolog E. What have we learnt from targeted anti-TNF therapy? Ann Rheum Dis. 2010;69: i97–i99. 10.1136/ard.2009.117143 19995756

[41] Chan JMK, Villarreal G, Jin WW, Stepan T, Burstein H, Wahl SM. Intraarticular Gene Transfer of TNFR:Fc Suppresses Experimental Arthritis with Reduced Systemic Distribution of the Gene Product. Mol Ther. 2002;6(6): 727–736. 10.1006/mthe.2002.0808 12498769

[42] Ashcroft GS, Horan MA, Ferguson MW. Aging alters the inflammatory and endothelial cell adhesion molecule profiles during human cutaneous wound healing. Lab Invest. 1998;78: 47–58. 9461121

[43] Lipschitz DA, Udupa KB. Influence of aging and protein deficiency on neutrophil function. J Gerontol. 1986;41: 690–694. 10.1093/geronj/41.6.690 3021843

[44] Wenisch C, Patruta S, Daxböck F, Krause R, Hörl W. Effect of age on human neutrophil function. J Leukoc Biol. 2000;67: 40–45. 10.1002/jlb.67.1.40 10647996

[45] Butcher SK, Chahal H, Nayak L, Sinclair A, Henriquez N V, Sapey E, et al Senescence in innate immune responses: reduced neutrophil phagocytic capacity and CD16 expression in elderly humans. J Leukoc Biol. 2001;70: 881–886. 11739550

[46] Song XY, Zeng L, Jin W, Thompson J, Mizel DE, Lei KJ, et al Secretory Leukocyte Protease Inhibitor Suppresses the Inflammation and Joint Damage of Bacterial Cell Wall-induced Arthritis. J Exp Med. 1999;190: 535–542. 10.1084/jem.190.4.535 10449524 PMC2195606

[47] Brüünsgaard H, Pedersen BK. Age-related inflammatory cytokines and disease. Immunol Allergy Clin North Am. 2003;23: 15–39. 10.1016/s0889-8561(02)00056-5 12645876

[48] Bruunsgaard H. Effects of tumor necrosis factor-alpha and interleukin-6 in elderly populations. Eur Cytokine Netw. 2002;13: 389–91. 12517724

[49] Kojima H, Inoue T, Kunimoto H, Nakajima K. IL-6-STAT3 signaling and premature senescence. JAK-STAT. 2013;2(4):e25763 10.4161/jkst.25763 24416650 PMC3876432

[50] Franceschi C, Campisi J. Chronic Inflammation (Inflammaging) and Its Potential Contribution to Age-Associated Diseases. Biol Sci Cite J as J Gerontol A Biol Sci Med Sci. 2014;69 10.1093/gerona/glu057 24833586

[51] Valacchi G, Lim Y, Belmonte G, Miracco C, Zanardi I, Bocci V, et al Ozonated sesame oil enhances cutaneous wound healing in SKH1 mice. Wound Repair Regen. 2011;19: 107–115. 10.1111/j.1524-475X.2010.00649.x 21134039

[52] Donato-Trancoso A, Monte-Alto-Costa A, Romana-Souza B. Olive oil-induced reduction of oxidative damage and inflammation promotes wound healing of pressure ulcers in mice. J Dermatol Sci. 2016;83: 60–69. 10.1016/j.jdermsci.2016.03.012 27091748

[53] Ishak WMW, Katas H, Yuen NP, Abdullah MA, Zulfakar MH. Topical application of omega-3-, omega-6-, and omega-9-rich oil emulsions for cutaneous wound healing in rats. Drug Deliv Transl Res. 2019;9: 418–433. 10.1007/s13346-018-0522-8 29667150

[54] Schanuel FS, Saguie BO, Monte-Alto-Costa A. Olive oil promotes wound healing of mice pressure injuries through NOS-2 and Nrf2. Appl Physiol Nutr Metab. 2019 [Epup ahed of print]. 10.1139/apnm-2018-0845 30901524

